# Polysaccharides from *Pseudostellaria heterophylla* modulate gut microbiota and alleviate syndrome of spleen deficiency in rats

**DOI:** 10.1038/s41598-022-24329-9

**Published:** 2022-11-23

**Authors:** Qing Xiao, Li Zhao, Chang Jiang, Yanjin Zhu, Jizhou Zhang, Juan Hu, Guozeng Wang

**Affiliations:** 1 Institute of Materia Medica, Fujian Academy of Chinese Medical Sciences, Fuzhou, Fujian People’s Republic of China; 2 Pharmacy Department, The Second Affiliated Hospital of Fujian University of Traditional Chinese Medicine, Fuzhou, Fujian People’s Republic of China; 3 College of Pharmacy, Fujian University of Traditional Chinese Medicine, Fuzhou, Fujian People’s Republic of China; 4 College of Biological Science and Engineering, Fuzhou University, Fuzhou, Fujian People’s Republic of China

**Keywords:** Medical research, Microbiology

## Abstract

*Pseudostellaria heterophylla*, also called Tai-zi-shen (TZS) in Traditional Chinese Medicine (TCM), is always used clinically to treat spleen deficiency symptoms. Polysaccharides in TZS have various pharmacological activities, including anti-diabetic, immune regulation, and myocardial protection. However, the relationship between the spleen-invigorating effects of TZS or its polysaccharides and intestinal flora are not clear. This study investigated the effects of TZS decoction (PHD) and polysaccharide (PHP) on immune function and intestinal flora in a rat model of spleen deficiency syndrome (SDS) induced by a decoction of raw rhubarb (RRD). PHD and PHP increased immune organ index, alleviated inflammatory cell filtration, and reduced the levels of pro-inflammatory cytokines in rats with spleen deficiency syndrome. In addition, the production of butyric acid was promoted in PHD and PHP groups. Moreover, 16S rRNA gene sequencing showed that PHD and PHP reduced the relative abundance of Firmicutes while increasing the one of Bacteroidetes; significantly increased the abundance of *Lactobacillus* and decreased the abundance of *Rombutsia*; and PHP significantly increased the abundance of *Alloprevotella*. And there was a significant positive correlation between the alleviation of SDS and short-chain fatty acids (SCFAs)-producing bacteria. These findings suggested PHD and PHP, especially PHP, has a potential to relieve spleen deficiency by reducing intestinal inflammation, modulating structure and composition of gut microbiota, and promoting the production of butyric acid.

## Introduction

In Traditional Chinese Medicine (TCM), “spleen” doesn’t refer to the anatomical spleen organ, but controls the digestion, absorption, transition and metabolism of dietary substances^1,2^. Spleen deficiency syndrome (SDS) is a common syndrome in TCM, caused by eating disorders, temperature discomfort, and overwork. Its clinical manifestations include diarrhea, weight loss, fatigue, and chills^1–4^. SDS is always closely related to digestive system diseases, gastrointestinal hormone disorders, immune deficiency and energy metabolism disorders in modern research^2,4–7^. The relationship between intestinal microbiota and SDS has been demonstrated by animal experiments and clinical studies^1,8–11^. In China, SDS is often treated with traditional Chinese medicines that strengthen the spleen and replenish Qi (spirit)^11–13^.

*Pseudostellaria heterophylla*, also called Tai-zi-shen (TZS) in Chinese, is the dry root tuber of *P. heterophylla* (Miq.) Pax ex Pax et Hoffim. TZS was first recorded in Běn Cǎo Cóng Xīn (in 1757) and has been widely used as both medicine and foodstuff^14^. As described in Pharmacopoeia of the People’s Republic of China, TZS has been found to invigorate the spleen and nourish Qi, promote fluid production and moisturize the lung^15^. TZS is always used in TCM to treat symptoms of spleen deficiency, such as diarrhea, loss of appetite and shortness of breath^16,17^. TZS and its extracts have been shown to reduce the incidence of spleen deficiency in mice, to improve the physical fitness of mice with spleen deficiency, and to significantly improve indices of mouse immune organs, such as the spleen and thymus, thus nourishing Qi and strengthening the spleen^16–18^. The main constituents of TZS include polysaccharides, cyclic peptides, amino acids, saponins and other chemical components, with polysaccharides being primarily responsible for the bioactivity of TZS^19^. Polysaccharides from TZS have various pharmacological activities, including anti-diabetic, immune regulatory, and myocardial protective activities^14,19–21^. However, little is known about the relationship between the spleen-invigorating effects of TZS and its polysaccharides and the intestinal flora.

The present study investigated the effects of *Pseudostellaria heterophylla* Decoction (PHD) and *Pseudostellaria heterophylla* Polysaccharide (PHP) in a rat model of SDS induced by a decoction of raw rhubarb administered by gavage. The effects of TZS on immune organ index, pro-inflammatory cytokines, short-chain fatty acids (SCFAs), and the structure and composition of intestinal flora were evaluated. The purpose of this study was to explore whether the ability of TZS to improve spleen deficiency syndrome was related to the effects of the polysaccharides on intestinal flora.

## Results

### The content and monosaccharide composition of the polysaccharide

The content of total sugar of the crude polysaccharide was 67.18% according to the glucose standard curve. The monosaccharide composition of the crude polysaccharide was analyzed based on the chromatographic peaks of glucose and galactose reference substance and samples. The results showed the polysaccharide was mainly composed of glucose and galactose, whose percentage content were about 44.25% and 14.43% (Fig. S1). Additionally, glucuronic acid, galacturonic acid, arabinose, mannose, xylose and xylose rhamnose were also detected in the crude polysaccharide.

### Effects of PHD and PHP on body weight and fecal moisture content

**Figure 1:**
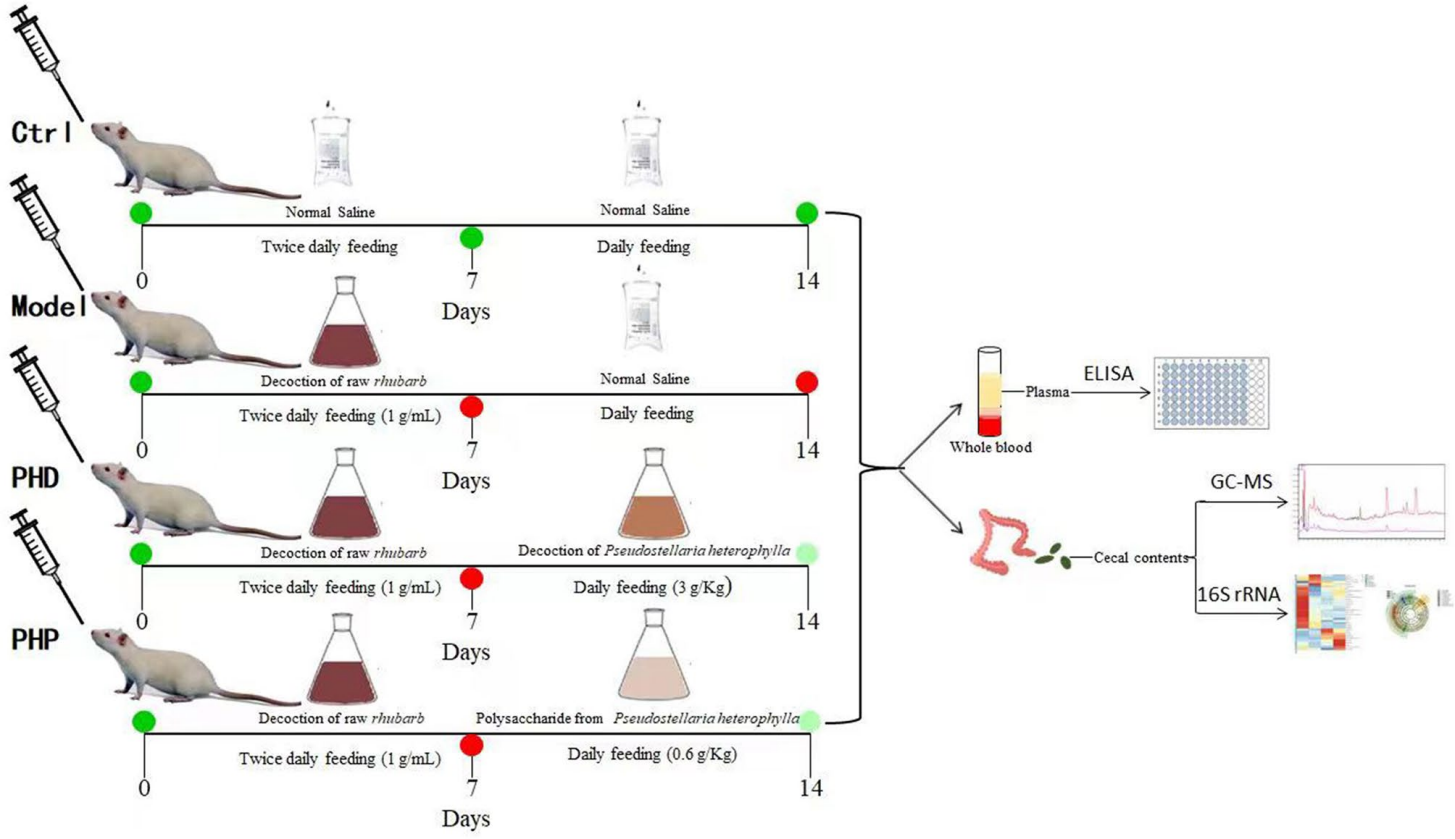
The Scheme of experimental plan and treatment procedure.

**Figure 2:**
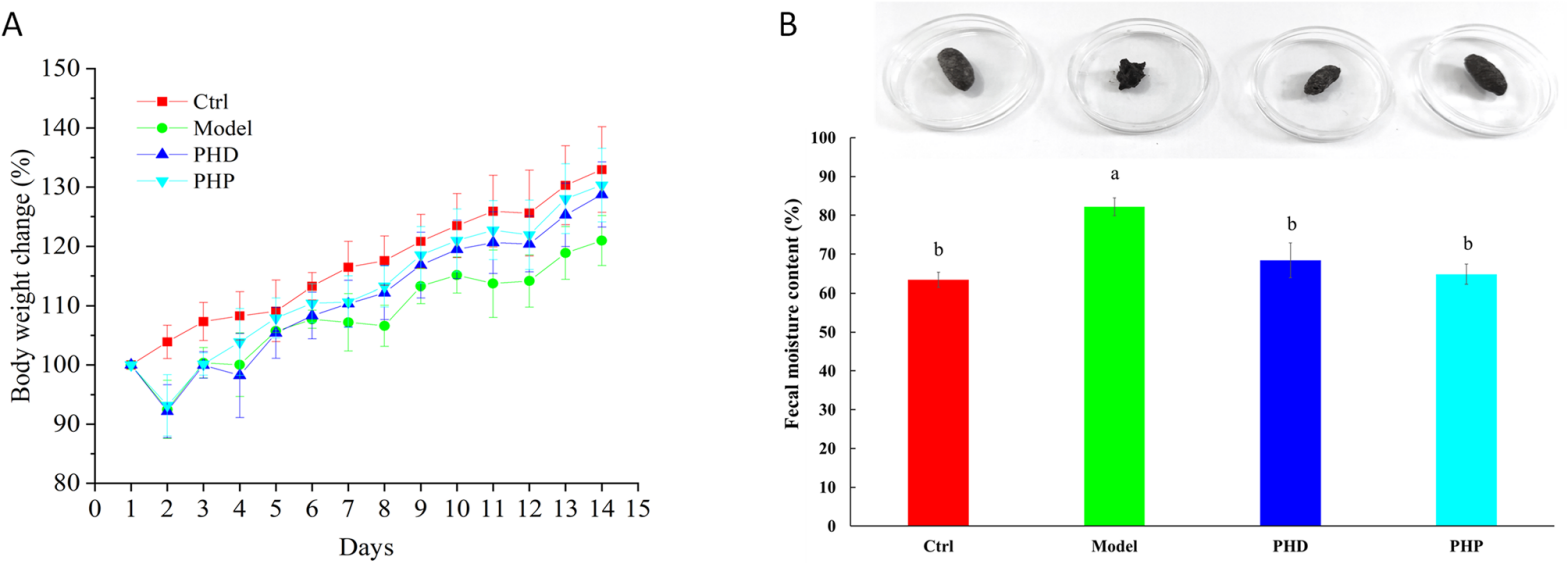
Effect of PHD and PHP on body weight of rats (**A**) and fecal moisture content (**B**). Values was presented as mean ± SD (n = 5). Significant differences (*p* < 0.05) were determined by one-way ANOVA and independent samples Kruskal–Wallis test using SPSS (version 33.0) software. Different lowercase letters (a and b) were significantly different at the level of *p* < 0.05*.*

Rats were divided into four groups; three groups were treated for 7 days with RRD to induce SDS and the fourth, Control (Ctrl) group was treated with saline alone (Fig. 1). The RRD-treated rats were subsequently treated on days 8–14 with a PHD, PHP, or saline (Model group), as illustrated in Table 1. And their body weights over 14 days were measured (Fig. 2A). The rate of weight gain over days 1–7 was higher in the Ctrl group than in the other three groups. Rats in the PHD, PHP, and Model groups showed symptoms of diarrhea, along with dull hair and an arched back, suggesting that these rats had SDS. The rate of weight gain over days 8–14 was significantly higher in rats treated with PHD and PHP than in the Model group, indicating that PHD and PHP could increase body weight in this rat model. In addition, the fecal moisture content of the Model group was significantly higher than that of the Ctrl (*p* < 0.05). And compared with the Model group, PHD and PHP significantly reduced the fecal moisture content (*p* < 0.05), as showed in Fig. 2B.

**Table 1:** Experimental plan and treatment procedure.

Time	Group			
	Ctrl	Model	PHD	PHP
1–7 days	Normal saline	RRD (10 g/Kg)	RRD (10 g/Kg)	RRD (10 g/Kg)
8–14 days	Normal saline	Normal saline	PHD (3 g/Kg)	PHP (0.6 g/Kg)

### Effects of PHD and PHP on gastrin in the plasma and amylase in the serum

Gastrin (GAS) is an important hormone for evaluating the physiological function of the gastrointestinal tract. As shown in Table 2, the level of GAS in the Model group was significantly decreased as compared with the Ctrl group (*p* < 0.05). Treatment with PHD and PHP resulted in significantly higher levels of GAS compared with Model group (*p* < 0.05). The level of amylase in the serum (AMS) is another parameter for assessing spleen deficiency. Comparisons showed that the concentration of AMS was significantly reduced in the Model group compared with the Ctrl group (*p* < 0.05). After medication with PHP, the level of AMS was significantly higher than in the Model group (*p* < 0.05). However, there was no significant difference between the Model and the PHD.

**Table 2:** Effects of PHD and PHP on gastrin (GAS) and amylase (AMS) in the serum.

Index	Group			
	Ctrl	Model	PHD	PHP
GAS (pg/mL)	911.20 ± 188.95^a^	688.02 ± 112.75^b^	1089.28 ± 109.34^a^	997.88 ± 161.48^a^
AMS (ng/mL)	15.47 ± 2.37^a^	6.67 ± 1.20^c^	10.25 ± 1.14 ^b^	11.95 ± 1.51 ^b^

Data are expressed as mean ± SD (n = 5). Different lowercase letters (a, b and c) were significantly different at the level of *p* < 0.05.

### Effects of PHD and PHP on immune organ index, histopathological changes and immunocytokines

The spleen and thymus are the main immune organs in mammals, with their weights being related to immune system functions. Comparisons showed that the spleen and thymus indices were significantly lower in the Model group than those in the Ctrl group (*p* < 0.05 each; Table 3). However, treatment with PHD or PHP resulted in significantly higher spleen and thymus indices than those in the Model group (*p* < 0.05 each).

**Table 3:** Effect of PHD and PHP on the immune organ index of rats (mg/g).

Index	Group			
	Ctrl	Model	PHD	PHP
Spleen	3.11 ± 0.13^a^	2.10 ± 0.46^b^	2.92 ± 0.14^a^	2.63 ± 0.08^a^
Thymus	2.15 ± 0.24^a^	1.48 ± 0.08^c^	1.75 ± 0.06^b^	1.82 ± 0.07^b^

Data are expressed as mean ± SD (n = 5). Different lowercase letters (a, b and c) were significantly different at the level of *p* < *0.05.*

**Figure 3:**
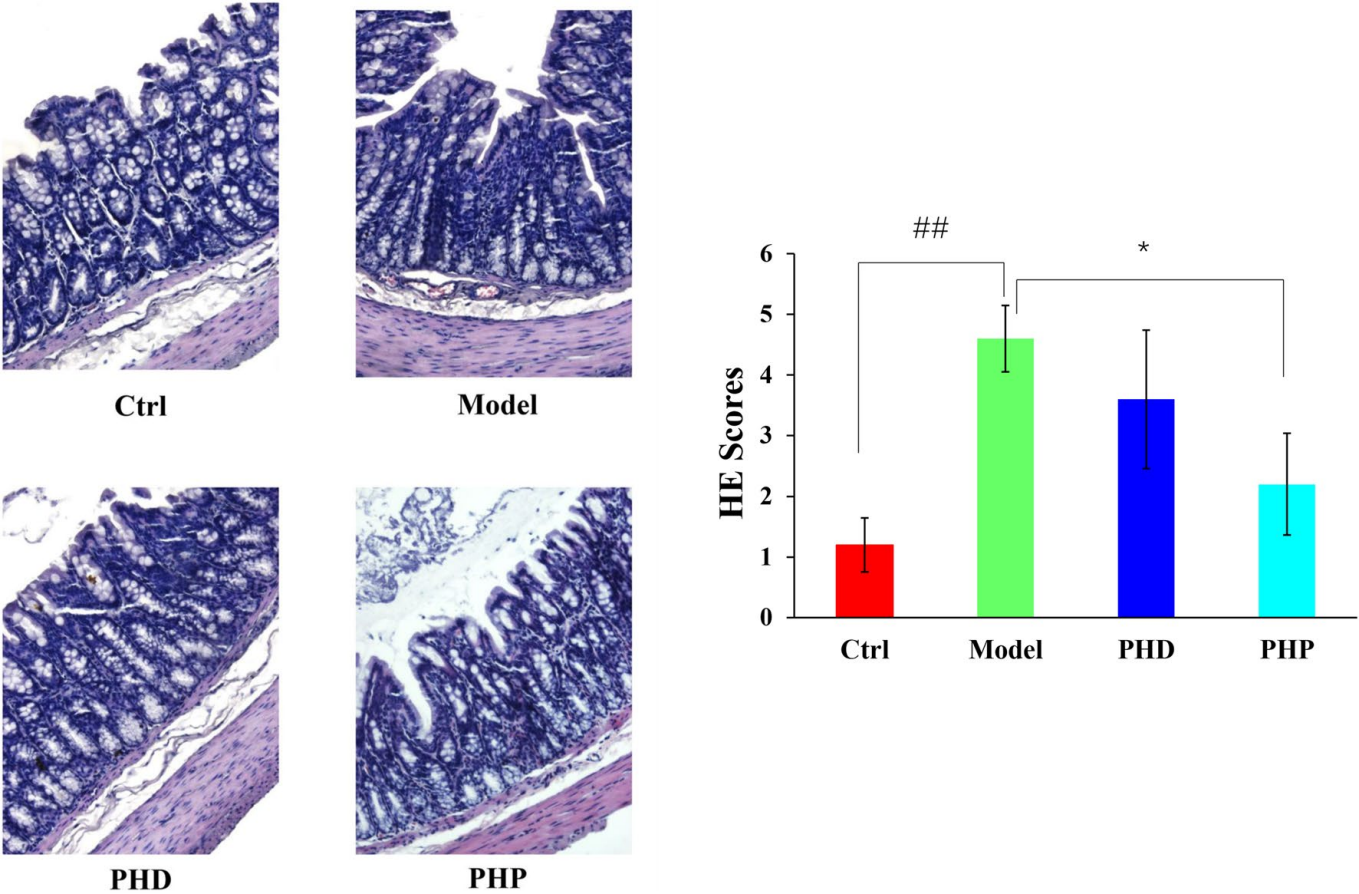
Representative images of the HE-stained sections (200 × magnification) from the colons in four groups. Values was presented as mean ± SD (n = 5). Significant differences (*p* < *0.05*) were determined by one-way ANOVA and independent samples Kruskal–Wallis test using SPSS (version 33.0) software.

The histopathological changes of the colons in four groups were analyzed by HE stained sections. As shown in Fig. 3, a large degree of inflammatory cell infiltration was observed in colons of rats in the Model group. In the PHD and PHP group, the degree of inflammatory cell infiltration was significantly reduced.

Immune system function has also been associated with the level of pro-inflammatory cytokines, such as IL-1β, IL-6 and TNF-α. The serum concentrations of IL-6 and TNF-α, as determined by ELISA, were significantly higher in the Model than in the Ctrl group (*p* < 0.05; Table 4). Intragastric administration of PHD and PHP significantly reduced the levels of IL-6 and TNF-α (*p* < 0.05). As to IL-1β, there was no significant difference among the four groups in the concentrations of IL-1β.

**Table 4:** Effect of PHD and PHP on the immune cytokines of rats.

Cytokine	Group			
	Ctrl	Model	PHD	PHP
IL-1β (pg/mL)	3.41 ± 0.35^a^	5.58 ± 1.74^a^	4.54 ± 0.85^a^	5.33 ± 1.63^a^
IL-6 (pg/mL)	1.58 ± 0.23^b^	2.50 ± 0.39^a^	1.83 ± 0.45^b^	1.85 ± 0.28^ab^
TNF-α (pg/mL)	8.78 ± 4.88^b^	18.56 ± 4.08^a^	13.37 ± 3.02^ab^	12.65 ± 3.27^ab^

Data are expressed as mean ± SD (n = 5). Different lowercase letters (a, b and c) were significantly different at the level of *p* < 0.05.

### Effects of PHD and PHP on the production of SCFAs

SCFAs, which are mainly produced through the digestion of non-starch polysaccharides by the intestinal microbiota, play critical roles in maintaining the balance between the gut and the host immune system^22,23^. Acetic acid, propionic acid and butyric acid were the most abundant SCFAs in the cecum of these rats, with the concentration of butyric acid in Model group was significantly lower than the one in Ctrl group (*p* < 0.05; Table 5). Although the levels of acetic acid, propionic acid were also reduced to a certain extent, there was no significant difference. Treatment with PHD and PHP significantly increased the level of butyric acid as compared with the Model group (*p* < 0.05 each), indicating that these agents could facilitate the ability of intestinal flora to produce more butyric acids.

**Table 5:** Effect of PHD and PHP on the short chain fatty acids (SCFAs) in the cecal contents of rats.

SCFAs	Group			
	Ctrl	Model	PHD	PHP
Acetic acid (μg/g)	1330.80 ± 217.97^a^	944.73 ± 115.64^a^	1223.02 ± 256.098^a^	1082.24 ± 187.49^a^
Propionic acid (μg/g)	904.83 ± 194.74^a^	686.01 ± 98.55^a^	873.93 ± 309.06^a^	901.72 ± 233.02^a^
Butyric acid (μg/g)	2551.75 ± 402.70^a^	1590.70 ± 375.37^b^	2142.51 ± 292.05^ab^	2252.89 ± 137.15^ab^

Data are expressed as mean ± SD (n = 5). Different lowercase letters (a, b and c) were significantly different at the level of *p* < *0.05.*

### Effects of PHD and PHP on cecal microbiota

**Figure 4:**
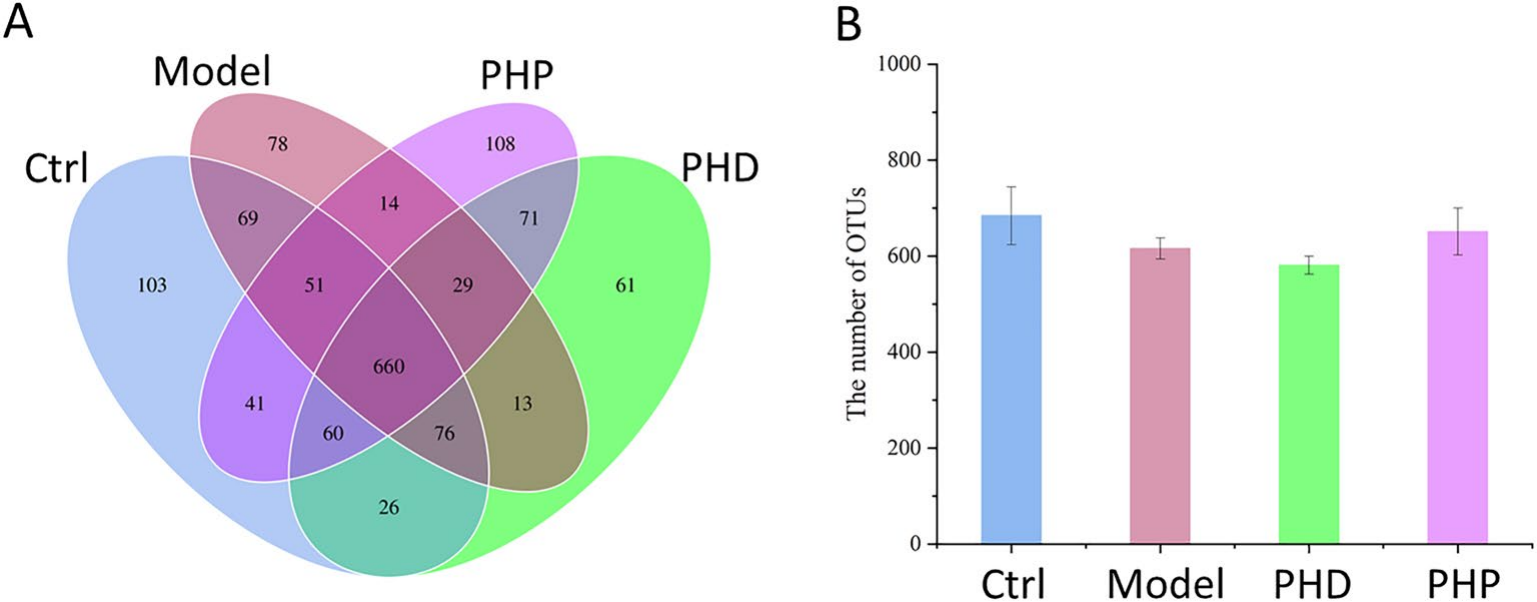
Venn diagram of common or endemic species among four groups: Ctrl group, Model group, PHD group and PHP group (**A**); histogram of OTUs in the above four groups (**B**). Values was presented as mean ± SD (n = 5).

The effects of PHD and PHP on the gut microbiota were evaluated by 16S rRNA gene sequencing analysis. A total of 1,266,939 effective tags were obtained by splicing, quality control and chimera filtering, including 302,596 tags in the Ctrl group, 324,644 in the Model group, 316,659 in the PHD group, and 323,040 in the PHP group (Table S1). Based on 97% agreement, the operational taxonomic units (OTUs) were clustered on the valid data of each sample. The Ctrl, Model, PHD, and PHP groups yielded totals of 684 ± 56, 616 ± 22, 581 ± 19, and 652 ± 48 OTUs, respectively, with a Venn diagram showing that 660 OTUs were shared by the four groups (Fig. 4A–B).

#### Diversity of gut microbiota

**Figure 5:**
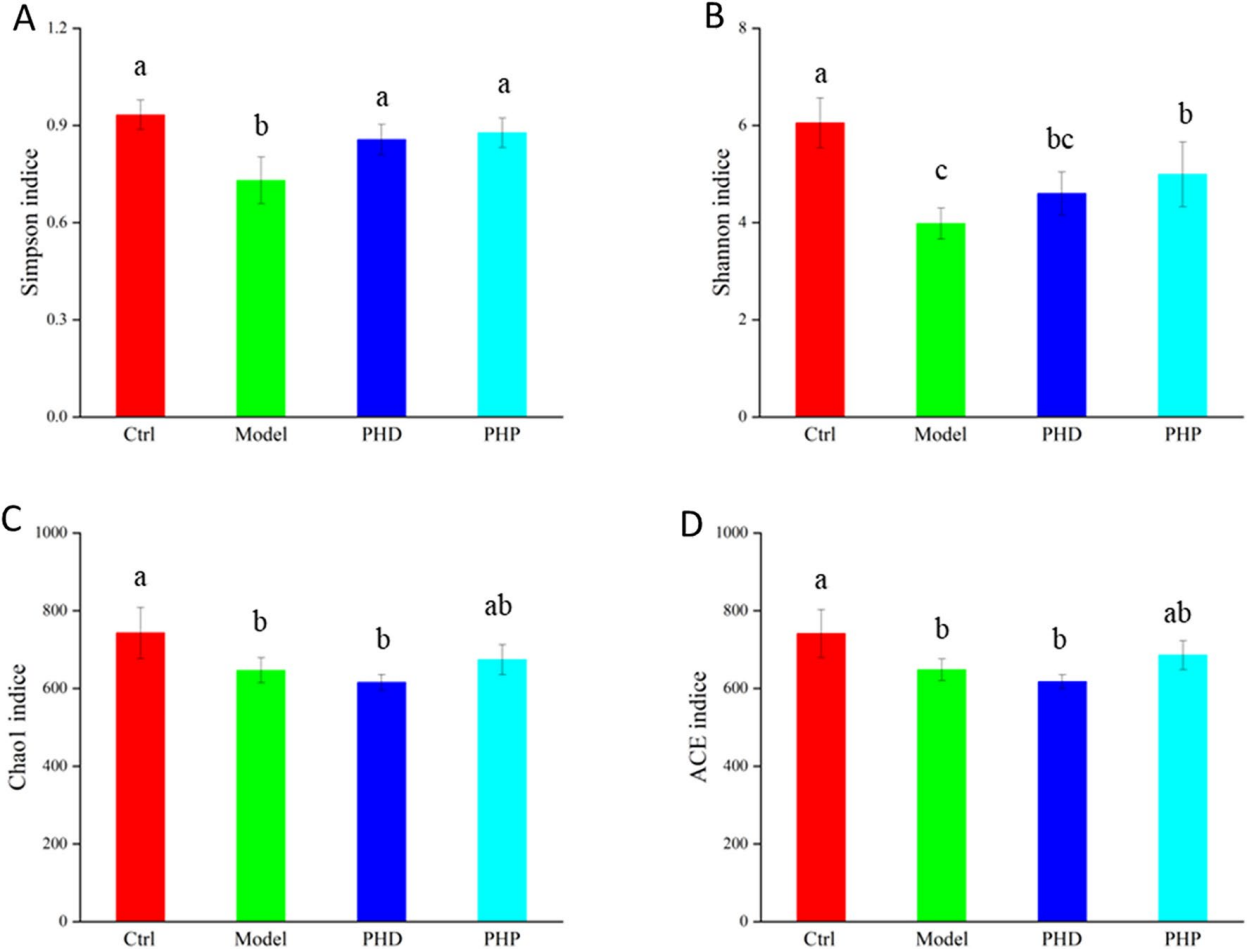
Effect of PHD and PHP on the α-diversity index (Simpson, Shannon, ACE and Chao1) of gut microbiota (**A**–**D**). Values was presented as mean ± SD (n = 5). Significant differences (*p* < *0.05*) were determined by one-way ANOVA and independent samples Kruskal–Wallis test using SPSS (version 33.0) software. Different lowercase letters (a, b and c) were significantly different at the level of *p* < *0.05.*

The diversity, richness and structural differences of microbial communities are frequently assessed by diversity analysis, including α- and β-diversity. α-Diversity was determined by the Simpson, Shannon, Chao1 and ACE indices, with community diversity determined by Simpson and Shannon indices and community richness by Chao1 and ACE indices. All four indices were significantly lower in the Model than in the Ctrl group (*p* < 0.05 each; Fig. 5A–D), indicating that RRD treatment reduced community diversity and richness. The Simpson and Shannon indices were higher in the PHD and PHP groups than in the Model group (*p* < 0.05), suggesting that PHD and PHP enhanced the diversity of gut microbiota. The Chao1 and ACE indices in the PHP group were also higher than those in the Model group, indicating that PHP treatment increased the richness of gut microbiota (*p* < 0.05). In contrast, the two indices in the PHD group did not differ significantly from those in the Model group, showing PHD intervention had no apparent effect on the richness of gut microbiota.

**Figure 6:**
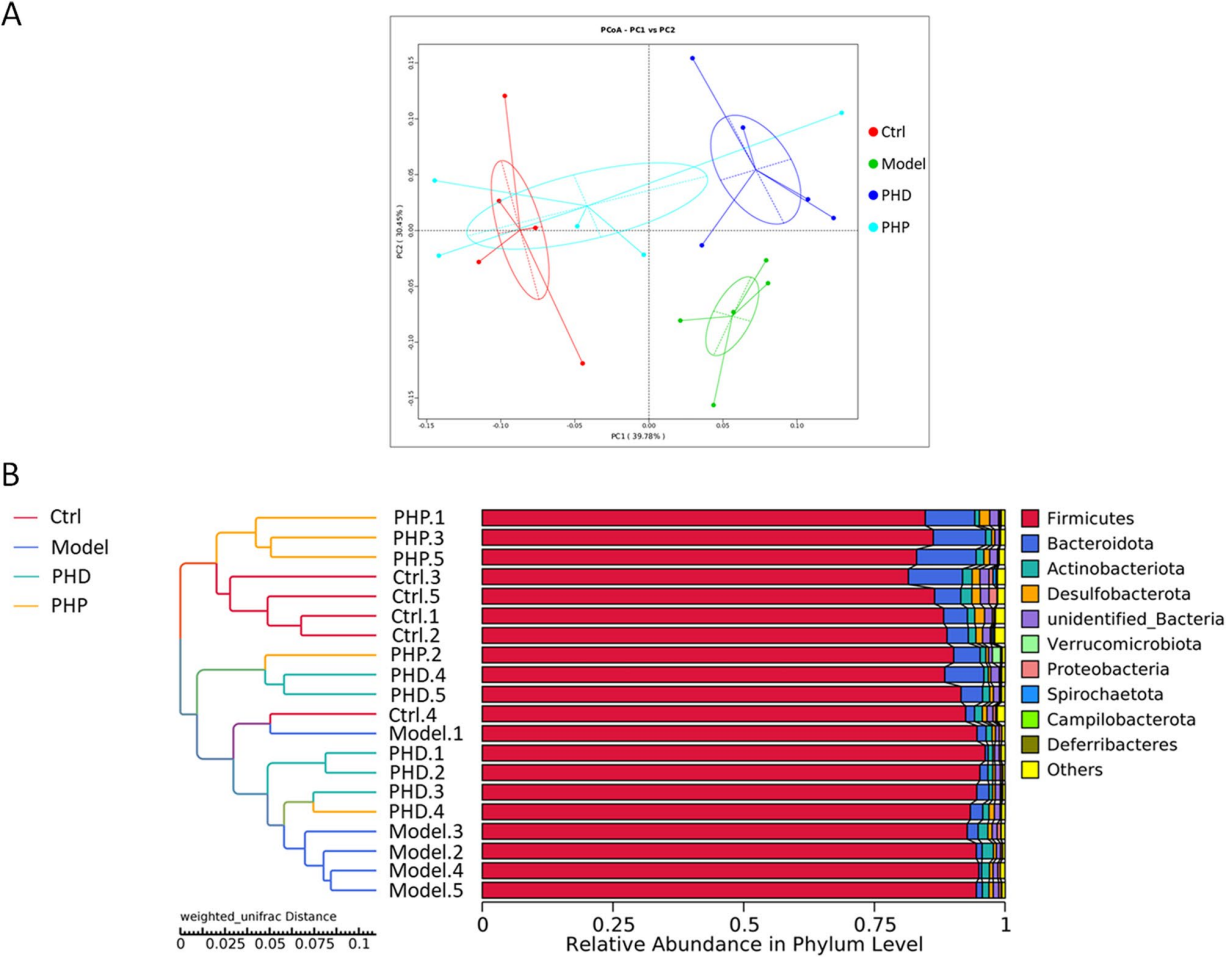
Effect of PHD and PHP on the β-diversity, which was assessed by PCoA analysis based on the unweighted UniFrac distances (**A**) and UPGMA based on the weighted UniFrac distance (**B**). Each plot represented one sample, n = 5.

β-Diversity was determined by PCoA, with a cluster tree evaluating the effects of PHD and PHP on the structure of gut microbiota. Weighted UniFrac PCoA of OTU abundance was performed to compare community similarities among the four groups. ANOSIM analysis indicated significant differences in community structure between groups, which were greater than that within-group (Fig. S2). The first principle coordinate (PC1), which accounted for 39.78% of the diversity, was the most important in distinguishing the Model group from the Ctrl group (Fig. 6A). Both the PHD and PHP groups were clearly distinguished from the Model group based on the second principle coordinate (PC2). Differences among samples were determined by a phylogenetic tree resulting from hierarchical clustering of weighted UniFrac distances. The Ctrl and Model groups were located in two branches of the cluster tree, with most of the samples PHP groups separated from the Model group (Fig. 6B). The group differences in β-diversity based on Weighted Unifrac were analyzed by Tuckey test (Fig. S3) and results showed significant differences between Ctrl group and Model group (*p* < 0.05), and between Model group and PHP group (*p* < 0.01). However, there were no significant differences between Model group and PHD group. Taken together, these findings indicated that treatment with PHP could alleviate the alteration of community structure induced by RRD.

#### Composition and abundance of gut microbiota

**Figure 7:**
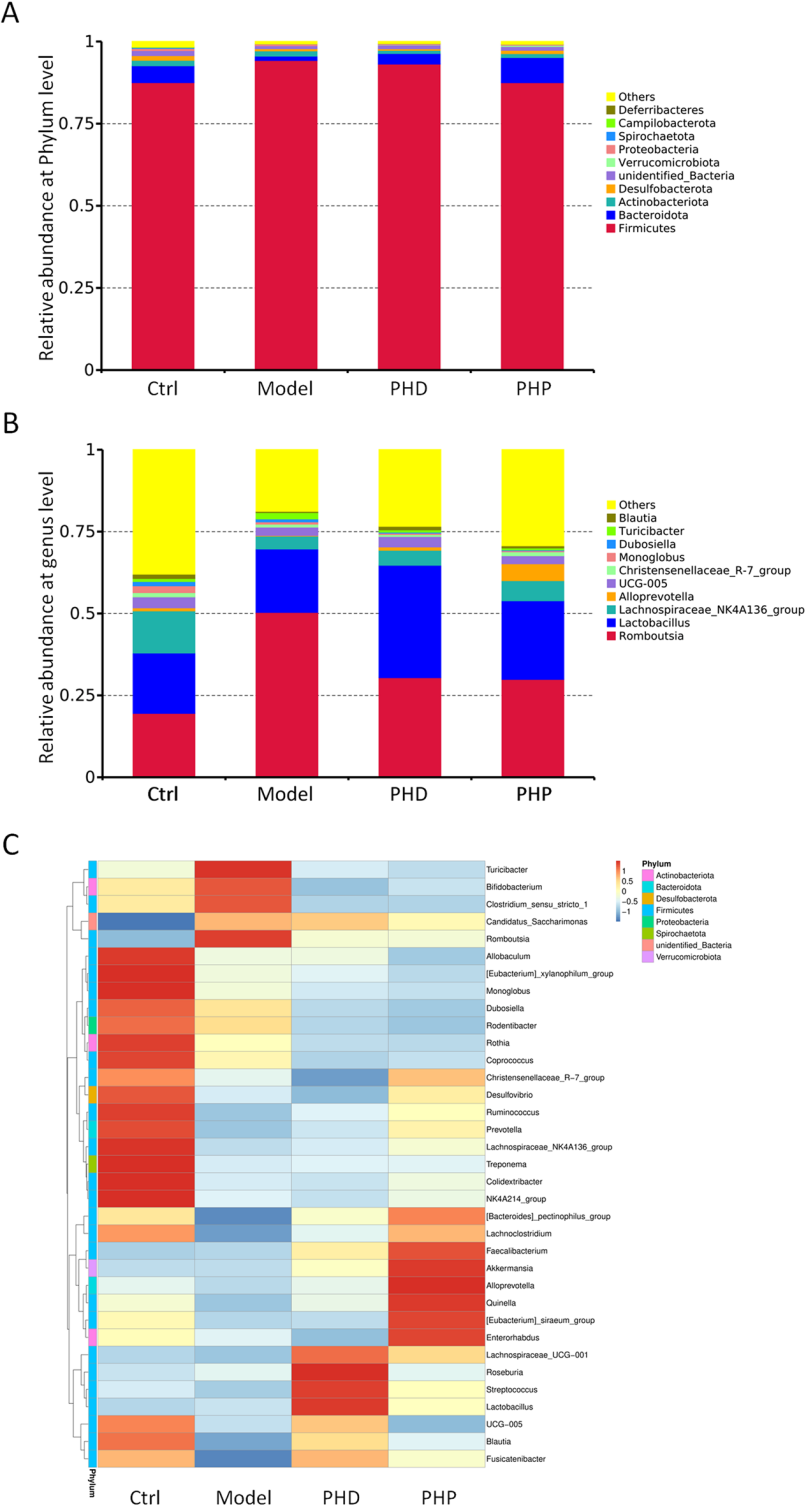
Effect of PHD and PHP on the composition and relative abundance of gut microbiota in the rats with spleen deficiency-induced diarrhea. (**A**) Relative abundance of gut microbiota at the level of phylum; (**B**) Relative abundance of gut microbiota at the level of genus; (**C**) Cluster heatmap of relative species abundance at genus level.

Ten phyla of gut microbiota compositions were detected at the phylum level in the four groups, with Firmicutes, Bacteroidetes, Actinobacteriota and Desulfobacterota being the dominant phyla. The distributions of these phyla differed among the four groups was different (Table S2). For example, the relative abundance of Firmicutes was higher and the relative abundance of Bacteroidetes lower in the Model group than in the Ctrl group (*p* < 0.05 each; Fig. 7A). Treatment with PHD and PHP significantly decreased the abundance of Firmicutes but increased the abundance of Bacteroidetes compared to the Model group (*p* < 0.05 each). And the value of Firmicutes/Bacteroidetes (F/B) ratio was significantly higher in the Model group than in the Ctrl group, but was reversed in the PHD and PHP groups, especially in the latter.

At the genus level, the predominant taxa were *Romboutsia*, *Lactobacillus*, Lachnospiraceae_NK4A136_group, *Alloprevotella*, UCG-005, *Monoglobus*, *Dubosiella*, *Blautia*, *Christensenellaceae*_R-7_group and *Turicibacter* (Fig. 7B,C). The relative abundance of *Romboutsia* was significantly higher, whereas the relative abundance of Lactobacillus was no significant change, and that of Lachonospiraceae_NK4A136_group and *Alloprevotella* were significantly lower, in the Model group than in the Ctrl group (Table S3). Treatment with PHD and PHP significantly reduced the relative abundance of *Romboutsia* (*p* < 0.05), while significantly increasing the levels of Lactobacillus (*p* < 0.05). In addition, the relative abundance *Alloprevotella* in the PHP group was significantly higher than in the Model group (*p* < 0.05).

**Figure 8:**
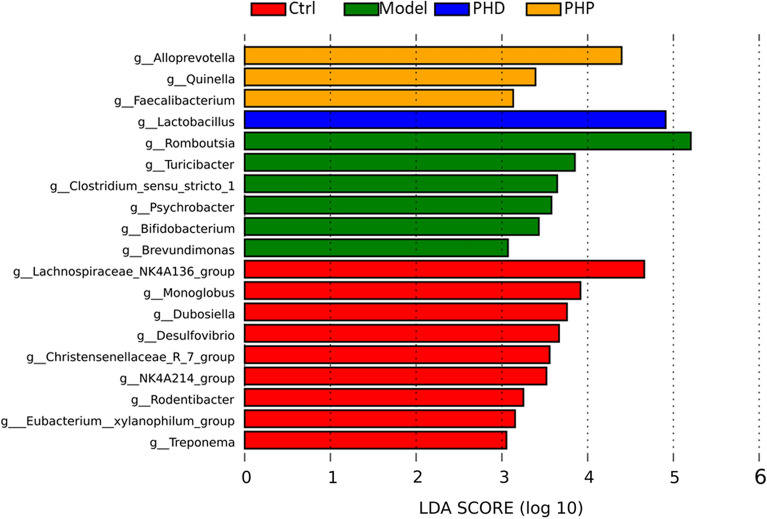
LDA score histogram of four groups (only taxa with LDA score higher than 3 were listed).

Biomarkers that differed significantly among these groups of rats were determined by linear discriminant analysis (LDA) effect size (LEfSe). An LDA score histogram showed that, when compared with the Ctrl group, the Model group was characterized by higher amounts of *Romboutsia*, *Turicibacter*, and *Clostridium_sensu*_stricto_1 and lower amounts of *Lachnospiraceae*_NK4A136_group, *Monoglobus, Alloprevotella*, *Desulfovibrio* and *Ruminococcus*, suggesting that the intestinal flora had been altered significantly (Fig. 8). The levels of *Alloprevotella* and *Fusicatenibacter* were higher in the PHD and PHP groups than in the Model group, with the abundance of the probiotic *Lactobacillus* being especially high. Moreover, the levels of other taxa, such as *Dialister*, *Quinella*, *Eubacterium_siraeum*_group and *Fusicatenibacter*, were markedly higher in the PHP than in the Model group. Taken together, these results indicate that PHD and PHP treatment could modulate specific communities of gut microbiota.

## Discussion

In clinical practice, TZS is mainly used to treat conditions such as spleen deficiency syndrome, fatigue, weakness, spontaneous sweating and thirst, and lung deficiency with cough and other symptoms^16,17,24^. Although TZS and its polysaccharides have been demonstrated to relieve symptoms of spleen deficiency, but few studies have assessed the relationships between the spleen-strengthening effects of TZS and intestinal flora. In this study, treatment with PHD and PHP increased AMS and GAS levels and immune organ index, but decreased the levels of IL-6 and TNF-α. Meanwhile, PHP treatment restored the structure and composition of intestinal microbiota, and promoted the butyric acid production.

Spleen deficiency is mainly manifested by weakened digestive function, abnormal secretion of gastrointestinal hormones, and decreased absorption in the small intestine^25^. Previous studies showed that weakened digestive function in SDS was affected by insufficient secretion of AMS^26^ and reduced activity of Na^+^-K^+^-ATPase^27^. In addition, SDS is also closely related to the secretion of gastrointestinal hormones^28^. Gastrin released from gastric sinus and duodenal G cells stimulates the secretion of gastric acid, pepsin, and bile and improves gastrointestinal motility^29^. In this study, higher levels of AMS and GAS were observed in the PHD and PHP groups as compared to the Model group, which indicated that PHD and PHP improved gastrointestinal digestive function.

Spleen deficiency is often accompanied by immune deficiency, which generally manifests as decrease in immune organ index, elevated levels of pro-inflammatory cytokines, and impaired intestinal barrier^5,11,30^. It has also been found in the present study that decreases in immune organ (spleen and thymus) index, increased levels of pro-inflammatory cytokines IL-6, and TNF-α and the significant inflammatory cell infiltration in colon in the Model group. Thymus and spleen, two important organs for immune cell differentiation and maturation, is closely related to the immune function^31,32^. The relative weights of thymus and spleen are considered as indicators of non-specific immunity^33^. In addition, TNF-α is a multifunctional cytokine secreted by macrophages that not only affects the systemic inflammatory response but also regulates the expression of other inflammatory cytokines. IL-6 is usually released in the early stages of inflammation and induce inflammatory response^30,34^. After intervention with PHD and PHP, the rats with SDS showed a significant increase in immune organ index and decrease in the levels of IL-6, and TNF-α, suggesting that PHD and PHP improved the immune function of SDS rats. Previous research by our group found that a purified polysaccharide from PHP (PF40) could enhance cell-mediated immunity by improving macrophage phagocytosis, splenocyte proliferation, NK cell activity and delayed-type hypersensitivity response^35^. This might be the mechanism for PHD and PHP enhancing immune function.

SDS has also been reported to be closely related to intestinal microbial disturbances, which refers to significant changes in the structure and composition of gut flora^1,10,11,28,36^. It has been reported that non-starch polysaccharides extracted from *Codonopsis Radix*, Radix Aconiti Lateralis Preparata and Sijunzi compound could regulate the structure and composition of intestinal flora and promote the reproduction of intestinal probiotics^3,5,37^. In present study, PHP not only have a positive effect on the diversity and abundance of probiotics, but also make the structure and composition of intestinal microorganisms close to those in the Ctrl group. After treatment with PHD and PHP, the Firmicutes (F) / Bacteroidetes (B) ration were decreased, with a greater decrease observed in the PHP group. The F/B ration is regarded as an important indicator of intestinal health, being positively correlated with the disorder of intestinal flora in various diseases, such as colitis, chemotherapy-type diarrhea and obesity^38,39^. In addition, the abundance of *Rombutsia* was reduced significantly, while the one of *Lactobacillus* increased significantly compared with the Model group. *Rombutsia* (belonging to Firmicutes) was reported to associate with protein metabolism in the gut^40^. *Lactobacillus* is a common probiotic that inhibiting the production of pro-inflammatory cytokines, and ameliorates intestinal dysfunction^41–43^. Several studies showed that the abundance of *Lactobacillus* was reduced in the gut flora of SDS rats^28,44^. And PHP treatment also markedly increased the abundance of *Alloprevotella*. These results implied PHD and PHP could modulate the intestinal flora in SDS rats.

The structure and function of gut microbial communities may contribute to inter- individual variation in cytokine response to microbial stimulations^45^. Aberrant immune responses accompanied by abnormal production of inflammatory cytokines are always closely linked to gut microbiome in large part by produce small molecules, like SCFAs, to mediate host-microbial interactions^46,47^. It has been shown that have intestinal microbial disturbances caused by SDS inhibited the production of SCFAs^7,48^. Based on the Spearman’s correlation, there was a significant positive correlation between the alleviation of SDS and SCFAs-producing bacteria (Fig. S4)*.* SCFAs are metabolites of the intestinal flora and enhance epithelial barrier function, improve intestinal permeability and inhibit inflammation^2,11,30^. Lachnospiraceae_NK4A136_group and *Alloprevotella* are the main producers of butyrate in the human intestine, which is the preferred energy source of colonic epithelial cells and is important for maintaining the function of the intestinal barrier^49,50^. In a word, PHP induced the intestinal flora to produce more butyric acid, might thereby depress the production of pro-inflammatory to regulate intestinal homeostasis.

## Conclusions

The present study showed that both PHD and PHP could alleviate SDS caused by a decoction of raw rhubarb, mainly by improving immune function, regulating intestinal flora, and promoting the production of SCFAs, with the effects of PHP being more significant. Intestinal flora plays important roles in the abilities of TZS to relieve SDS, with the polysaccharides in TZS being response for these effects. Because the polysaccharides used in this experiment are crude polysaccharides from TZS, it is unclear whether these effects are dependent on the molecular weights and chemical structures of polysaccharides. Purified polysaccharide from TZS would be used to demonstrate the role of gut microbiota on ameliorating SDS with more SDS models in future.

## Materials and methods

### Preparation of the decoction of raw rhubarb (Da-huang)

Desiccated roots of raw rhubarb were immersed in water at a solid:liquid ratio of 1:10 (m/v) for 30 min and heated to slight boiling for 1 h. The mixture was filtered with gauze and concentrated to 1/10 of the original volume by evaporation at 80 °C. This decoction of raw rhubarb (1 g/mL) was stored at 4 °C for further use.

### Preparation of the decoction of TZS

*Pseudostellaria Heterophylla* (TZS) was produced in Zherong County, Fujian Province, China. Dried roots of TZS were cut into small pieces of 0.3–0.5 cm. The pieces were immersed in water at a solid:liquid ratio of 1:5 (m/v) for 0.5 h and heated to slight boiling for 1 h. The procedure was repeated, and the mixture was filtered with gauze and concentrated to 2/5 of the original volume. This decoction of TZS (0.5 g/mL) was stored at 4 °C for further use.

### Preparation of crude polysaccharides

The desiccated roots of TZS were ground, passed through a 20-mesh sieve, and immersed in petroleum ether at a solid:liquid ratio of 1:2 (m/v). The mixture was extracted twice under reflux at 60 °C for 2 h. The non-fat powder was extracted twice with water at a solid:liquid ratio of 1:10 (m/v) under reflux at 100 °C for 2 h. After cooling to room temperature, the mixture was filtered and centrifuged at 5000 rpm for 15 min to remove solid materials. The supernatant was concentrated to 1/4 the original volume in a thermostatic water bath at 100 °C. The concentrated liquid was deproteinized by the Sevege method with n-butyl alcohol and chloroform, at a liquid:butyl alcohol:chloroform ratio of 25:5:1 (v/v/v). The mixture was shaken for 15 min and centrifuged at 5000 rpm for 5 min. The supernatant was mixed with four volumes of absolute ethanol and incubated overnight. The under-layer precipitate was dissolved in water and freeze-dried to obtain crude polysaccharide powder from *Pseudostellaria heterophylla*. The content of polysaccharide was determined by phenol–sulfuric acid method with D-glucose as standard^51^. The monosaccharide composition was analyzed according to the previous method^14^. Polysaccharide was hydrolyzed with trifluoroacetic acid (2 M) at 110 °C for 6 h. The hydrolysate was derivatizated with 1-phenyl-3-methyl-5-pyrazolinone (PMP) at 70 °C for 1.5 h. And high performance liquid chromatography (HPLC) was used to determine the monosaccharides of PHP.

### Animals and experimental design

Twenty male SD rats aged 6–8 weeks and weighing 200 ± 20 g (SLAC Laboratory Animal Co. Ltd.; Shanghai, China) were housed in a 12 h light/dark cycle room at auto-regulated temperature (20–25 °C) and humidity (50 ± 10%). All rats had unrestricted access to regular rat chow and water. The feed for rats was purchased from Beijing HFK Bioscience Co., LTD and its composition was listed in Table S4. After 7 days of acclimatization, the rats were randomly divided into four groups of five rats each, designated the Ctrl, Model, PHD, and PHP groups. Rats were treated for 14 days as illustrated in Table 1. Animal protocols were reviewed and approved by Ethics Committee on experimental animals of Fujian Academy of Chinese Medical Sciences (No. FJATCM—IAEC2020019). All experiments and methods were performed in accordance with the relevant guidelines and regulations. And the animal experiments was conducted in compliance with the ARRIVE (Animal Research: Reporting of In Vivo Experiments) guidelines^52^.

### Induction of SDS and treatment protocol

SDS was induced in rats by oral gavage with the RRD (1 g/mL) twice daily on days 1–7. Rats in the PHD group received a TZS decoction (3 g/kg) and rats in the PHP group received a crude polysaccharide solution of TZS (0.6 g/kg) twice daily by oral gavage on days 8–14. The dose of PHD for rats (3 g/kg) was calculated from the maximum clinical dose of TZS (30 g) recommended in the Pharmacopoeia of the People’s Republic of China. And the dose of PHP (0.6 g/kg) was 21.71% in TZS based on the content of polysaccharide, which was determined by phenol–sulfuric acid method with D-glucose as standard. Rats were weighed daily. The stools of each rat were collected. Abdominal aortic blood samples were collected 24 h after the last dose, and serum was collected by centrifugation. The cecal contents of each rat were also collected, immediately frozen in liquid nitrogen, and stored at − 80 °C for further analysis.

### Evaluation of fecal moisture content

The collected stools were dried to measure fecal moisture content (FMC) using the following formula:

$$FMC \left(\%\right)=\frac{wet \,\,mass \,\,before \,\,drying-dry \,\,mass \,\,after \,\,drying }{wet \,\,mass \,\,before \,\,drying}\times 100\%$$

### Histopathological analysis

The colons were washed with saline and fixed immediately in 4% paraformaldehyde solution for 24 h. Tissue samples were dehydrated in alcohol with graded concentrations, embedded in paraffin. and then sectioned using a microtome to obtain 5 μm-thick sections. And the sections were stained with hematoxylin and eosin (HE) and imaged by using a fluorescent inverted microscope (DMIL LED, Leica, Germany).

### Determination of pro-inflammatory cytokine concentrations by ELISA

The levels of AMS were determined using enzyme-linked immunosorbent assay (ELISA) kit (Product code number: ml059302, Shanghai Enzyme-linked Biotechnology Co., Ltd, Shanghai, China). The concentration of GAS and the level pro-inflammatory cytokines IL-1β, IL-6, and TNF-α in serum were quantified using ELISA kits respectively (Product code numbers: E-EL-R0639C, E-EL-R0012C, E-EL-R0015C, E-EL-R0019C, Elabscience Biotechnology Co., Ltd, Shanghai, China) according to the manufacturer’s instructions.

### Determination of short chain fatty acids contents by GC–MS

1 mL ultrapure water was added to a sample of cecal contents (50 ± 1 mg), vortex mixing for 10 s. The mixture was homogenized in ball mill for 4 min at 40 Hz and ultrasound in ice water for 5 min. This procedure was repeated three times. After being centrifuged (4 °C, 5000 rpm, 20 min), the supernatant (0.8 mL) was transferred into a mixed solution containing 0.1 mL 50% H_2_SO_4_ and 0.8 mL 2-Methylvaleric acid (25 mg/L stock in methyl tert-butyl ether) as internal standard, vortex mixing for 10 s, oscillating for 5 min, and ultrasound in ice water for 10 min. After being centrifuged (4 °C, 10,000 rpm, 15 min) and standing at -20 °C for 30 min, the supernatant was obtained to determine the fatty acids in cecal contents by GC–MS analysis.

An HP-FFAP capillary column and an Agilent 7890B gas chromatograph system coupled to a Agilent 5977B mass spectrometer were used to perform GC–MS. A 1-μL aliquot of each analyte was injected in split mode (5:1). Helium was used as the carrier gas, the front inlet purge flow was 3 mL min^−1^, and the gas flow rate through the column was 1 mL min^−1^. The initial temperature was kept at 80 °C for 1 min; increased to 200 °C at a rate of 10 °C min^−1^, maintained at 200 °C for 5 min; increased to 240 °C at a rate of 40 °C min^−1^, and maintained at 240 °C for 1 min; The injection, transfer line, quad and ion source temperatures were 240 °C, 240 °C, 230 °C and 150 °C, respectively. The energy was -70 eV in electron impact mode. Mass spectrometry data were acquired in Scan/SIM mode with an m/z range of 33–150 after a solvent delay of 4.0 min.

The concentrations of short chain fatty acids were calculated by the following formula:

$${C}_{\left(con\right)}=\frac{{C}_{s}\times {V}_{1}\times {V}_{3}}{M\times {V}_{2}}\times 1000$$

where ${C}_{(con)}$ is the content of the target compound in the cecal contents (ug g^−1^), ${C}_{s}$ is the concentration of the target compound in the supernatant for GC–MS analysis (mg L^−1^), ${V}_{1}$ is the volume of the internal standard solution (mL), ${V}_{2}$ is the volume of supernatant (mL), ${V}_{3}$ is the volume of ultrapure water (mL), $\mathrm{M}$ is the weight of cecal contents (mg).

### Intestinal microbiota analysis by 16S rRNA gene sequencing

Metagenomic DNA was extracted from each cecal sample using a DNeasy Powersoil kit (Qiagen, Hilden, Germany) in accordance with the manufacturer’s instructions. The V3–V4 hypervariable region of the 16S rRNA gene was amplified from the metagenomic DNA with the primers: 314F (5′-CCTAYGGGRBGCASCAG-3′) and 806R (5′-GGACTACNNGGGTATCTAAT-3′). The PCR products were purified using Qiagen Gel Extraction Kit (Qiagen, Hilden, Germany). A sequencing library was generated, assessed and sequenced on an Illumina NovaSeq 6000 platform (250 bp paired-end reads). Paired-end reads were merged using FLASH^53^ (version 1.2.7, http://ccb.jhu.edu/software/FLASH/). Quality filtering on the raw tags was performed under specific filtering conditions to obtain the high-quality clean tags according to the QIIME^54^ (version 1.9.1, http://qiime.org/index.html) quality controlled process. The tags were compared with the reference database (Silva database, using UCHIME algorithm (UCHIME http://www.drive5.com/usearch/manual/uchime_algo.html)^55,56^ to detect chimera sequences, and then the chimera sequences were removed and the effective tags were finally obtained. Operational taxonomic units (OTUs) were clustered with a similarity of 97% using UPARSE software^57^ (version 7.0.1001) and annotated using QIIME software. Alpha diversity indices, Chao1, Shannon, Simpson and ACE, were calculated with QIIME and analyzed with R software (Version 2.15.3). Beta diversity on both weighted and unweighted unifrac was calculated by QIIME. Principal Coordinate Analysis (PCoA) was performed to get principal coordinates and visualize from complex, multidimensional data. Weighted Pair-group Method with Arithmetic Means (UPGMA) Clustering was performed as a type of hierarchical clustering method to interpret the distance matrix using average linkage and was conducted by QIIME software. LDA Effect Size (LEfSe) analysis was performed with LEfSe software (Version 1.0) to identify the species with significant differences between groups^58^.

### Statistical analysis

Experimental data were reported as the mean ± standard deviation (SD). Differencesbetween groups were analyzed by one-way ANOVA and independent samples Kruskal–Wallis test using SPSS (version 33.0) software, with *p* values < 0.05 considered statistically significant. Different lowercase letters (a and b) were significantly different at the level of *p* < 0.05, which were compared by one-way ANOVA followed by Bonferroni LSD test.

## Supplementary Information

Supplementary Information.

## Data Availability

The datasets generated during the current study are available in the online repository. The names of the repository and accession numbers can be found below: https://www.ncbi.nlm.nih.gov/, ON380011-ON380018.
