# Supplementary material for: Polysaccharides from Pseudostellaria heterophylla modulate gut microbiota and alleviate syndrome of spleen deficiency in rats
Source: Sci Rep. 2022 Nov 23;12:20217. doi: 10.1038/s41598-022-24329-9 (PMC9684442; doi:10.1038/s41598-022-24329-9)
Supplement: Supplementary file 1 — Supplementary Information. [file 41598_2022_24329_MOESM1_ESM.docx]

**Supporting Information for**

**Polysaccharides from Pseudostellaria heterophylla Modulate Gut Microbiota and Alleviate Syndrome of Spleen Deficiency in Rats**

Qing Xiao^#1^, Li Zhao^#1^, Chang Jiang^1^, Yanjin Zhu^1^, Jizhou Zhang^1^, Juan Hu^*1,2,3^, Guozeng Wang*^4^

^1^Institute of Materia Medica, Fujian Academy of Chinese Medical Sciences, Fuzhou, Fujian, P.R. China

^2^Pharmacy Department, The Second Affiliated Hospital of Fujian University of Traditional Chinese Medicine, Fuzhou, Fujian, P.R. China

^3^College of Pharmacy, Fujian University of Traditional Chinese Medicine, Fuzhou, Fujian, P.R. China

^4^College of Biological Science and Engineering, Fuzhou University, Fuzhou, Fujian, P.R. China

*** Correspondence:**Juan Hu, Guozeng Wang

[huj@fjtcm.edu.cn](mailto:huj@fjtcm.edu.cn), [wanggz@fzu.edu.cn](mailto:wanggz@fzu.edu.cn)

**Figure S1** The chromatograms of (A) glucose and galactose reference substances and (B) crude samples with PMP hydrolysis (1 and 2 refers to gluctose and galactose)

**
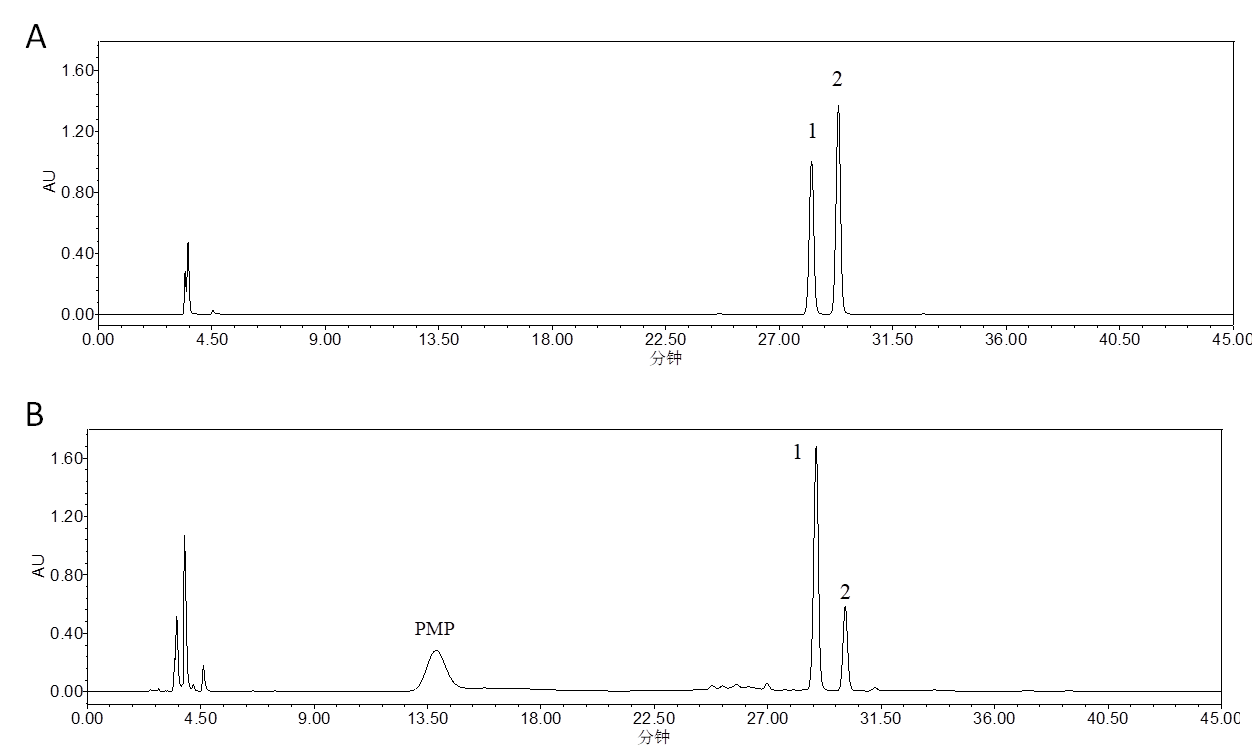
**

**Figure S2** Significance differences in community stricter between groups based on ANOSIM analysis.

**
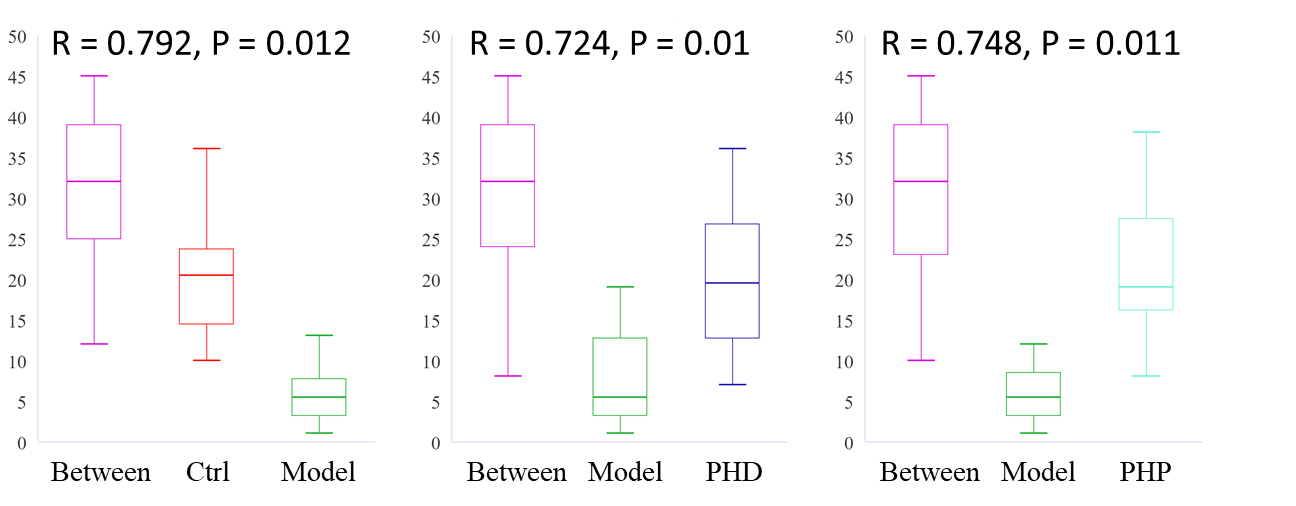
**

**Figure S3** Boxplot of β-diversity based on Weighted Unifrac.

**
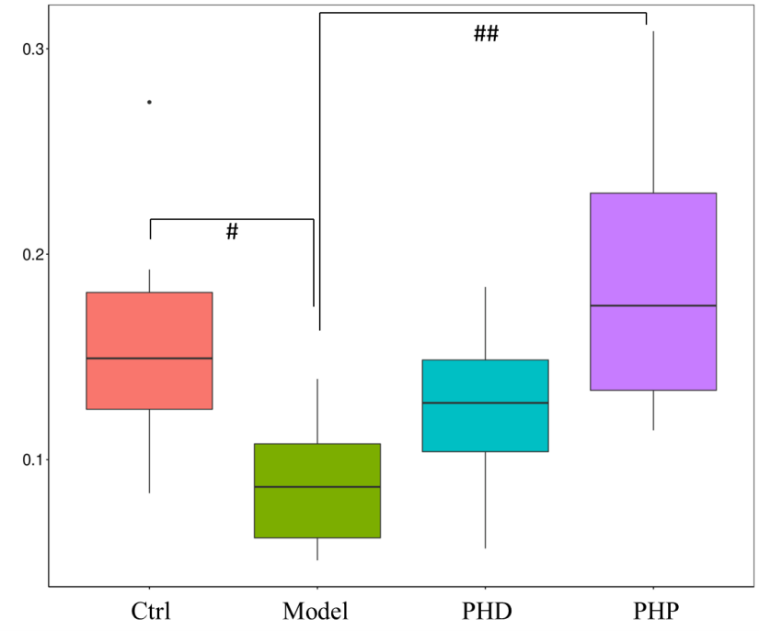
**

**Figure S4** The relationship between intestinal flora and indicators of SDS based on Spearman correlation analysis.

**
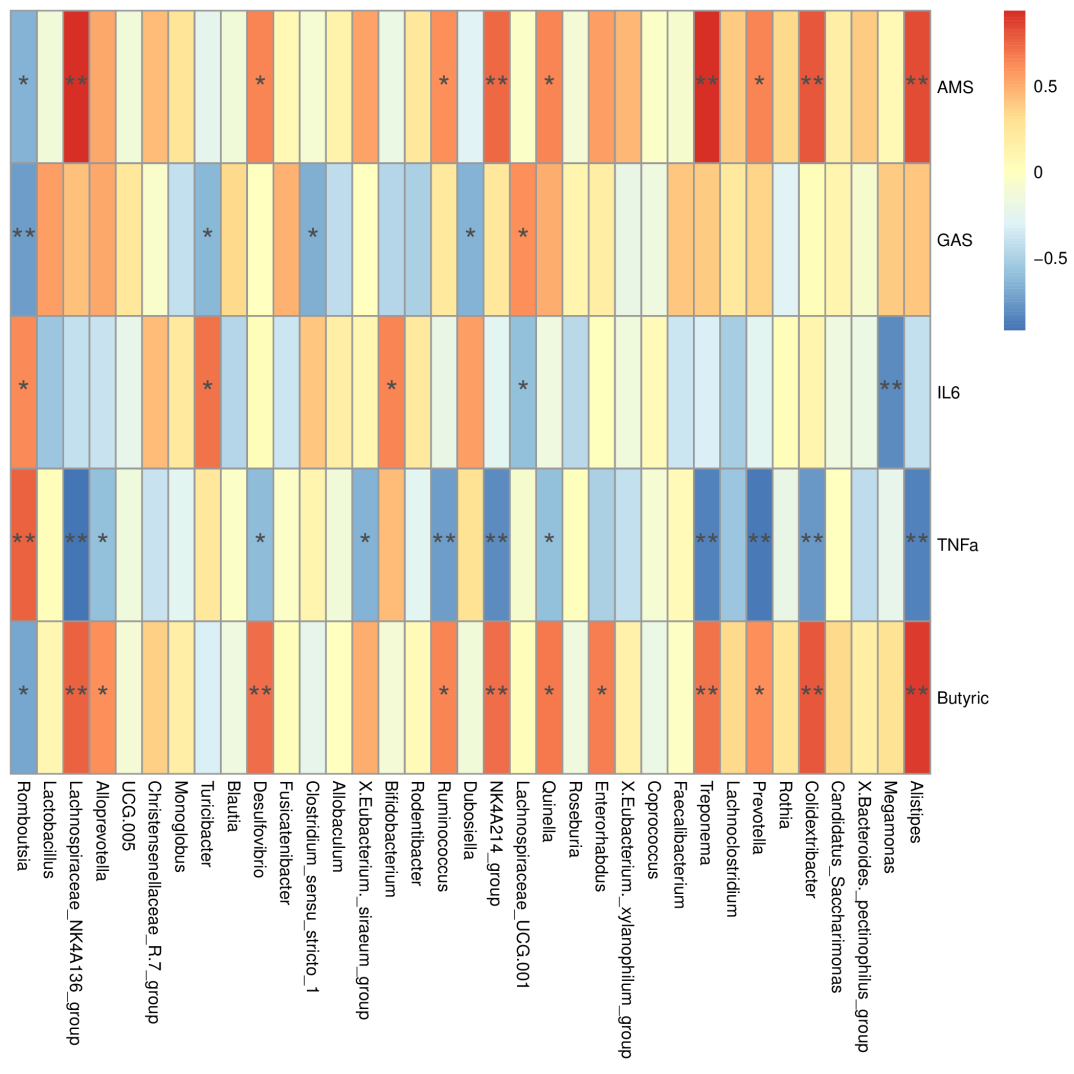
**

**Table S1** Data preprocessing statistics and quality control.

| Group | Sample | Raw PE | Raw Tags | Clean Tags | Effective Tags |
| --- | --- | --- | --- | --- | --- |
| Control | 1 | 96039 | 86320 | 84229 | 59260 |
|  | 2 | 105330 | 95195 | 92209 | 63188 |
|  | 3 | 90145 | 82843 | 80980 | 58159 |
|  | 4 | 100743 | 95000 | 92555 | 60745 |
|  | 5 | 103520 | 96851 | 94440 | 61244 |
| Model | 1 | 97401 | 85911 | 83641 | 60299 |
|  | 2 | 103744 | 88547 | 85949 | 63322 |
|  | 3 | 101812 | 90863 | 88147 | 63388 |
|  | 4 | 113222 | 99958 | 97024 | 68829 |
|  | 5 | 109105 | 100163 | 97487 | 68806 |
| PHD | 1 | 100370 | 85984 | 83,171 | 61,400 |
|  | 2 | 111,514 | 99,801 | 98,871 | 66,663 |
|  | 3 | 105,519 | 92,926 | 89,331 | 62,798 |
|  | 4 | 101,209 | 91,497 | 87,642 | 61,759 |
|  | 5 | 106,502 | 92,170 | 86,901 | 64,039 |
| PHP | 1 | 101,627 | 87,810 | 83,499 | 64,102 |
|  | 2 | 101,921 | 87,071 | 83,569 | 61,377 |
|  | 3 | 106,428 | 91,684 | 87,831 | 68,427 |
|  | 4 | 100,391 | 86,905 | 83,437 | 63,198 |
|  | 5 | 109,844 | 95,437 | 91,770 | 65,936 |

**Table S2** The relative abundance of dominant phyla at phylum level.

| Taxonomy | Ctrl group | Model group | PHD group | PHP group |
| --- | --- | --- | --- | --- |
| Firmicutes | 87.52±4.01^b^ | 94.26±0.89^a^ | 93.20±3.15^ab^ | 87.52±4.15^b^ |
| Bacteroidota | 5.16±3.20^ab^ | 1.34±0.59^b^ | 3.21±2.73^ab^ | 7.65±3.77^a^ |
| Actinobacteriota | 1.64±0.32^a^ | 1.58±0.41^a^ | 0.93±0.26^b^ | 1.16±0.20^ab^ |
| Desulfobacterota | 1.44±0.38^a^ | 0.69±0.13^b^ | 0.50±0.14^b^ | 1.03±0.58^ab^ |

Data are expressed as mean ± SD (n=5). Different lowercase letters (a, b and c) were significantly different at the level of p<0.05.

**Table S3** The relative abundance of dominant taxa at genus level.

| Taxonomy | Ctrl group | Model group | PHD group | PHP group |
| --- | --- | --- | --- | --- |
| *Romboutsia* | 19.54±11.38^b^ | 50.37±7.30^a^ | 30.45±9.53^b^ | 29.91±7.67^b^ |
| *Lactobacillus* | 18.43±7.07^b^ | 19.37±6.79^ab^ | 34.32±5.75^a^ | 24.01±11.71^ab^ |
| Lachnospiraceae_NK4A136_group | 12.86±1.77^a^ | 3.94±0.38^b^ | 4.58±1.96^b^ | 6.15±1.94^b^ |
| *Alloprevotella* | 0.94±0.90^b^ | 0.18±0.32^b^ | 1.01±0.73^b^ | 5.15±2.88^a^ |

Data are expressed as mean±SD (n=5). Different lowercase letters (a, b and c) were significantly different at the level of p<0.05.

| Protein | ≥180 g | His | ≥4.0 g | vitK | ≥3.0 mg | vitC | - |
| --- | --- | --- | --- | --- | --- | --- | --- |
| Fat | ≥40 g | Trp | ≥1.9 g | vitB1 | ≥8.0 mg | Mg | ≥2.0 g |
| Fiber | ≤50 g | Phe + Tyr | ≥11 g | vitB2 | ≥10 mg | K | ≥5.0 g |
| Ash | ≤80 g | Thr | ≥6.5 g | vitB6 | ≥6.0 mg | Na | ≥2.0 g |
| Moisture | ≤100 g | Lue | ≥14.4 g | vitB3 | ≥45 mg | Fe | ≥100 mg |
| Ca | 10-18 g | Ile | ≥7.0 g | vitB5 | ≥17 mg | Mn | ≥75 mg |
| P | 6-12 g | Val | ≥8.4 g | Folate | ≥4.0 mg | Cu | ≥10 mg |
| Lys | ≥8.2 g | vitA | ≥7000 IU | Biotin | ≥0.1 mg | Zn | ≥30 mg |
| Met + Cys | ≥5.3 g | vitD | ≥800 IU | vitB12 | ≥0.02 mg | I | ≥0.5 mg |
| Arg | ≥9.9 g | vitE | ≥60 IU | Choline | ≥1250 mg | Se | 0.1-0.2 mg |

**Table S4** Diet composition for rats (calculated per kg of feed).
